# Supplementary material for: Protocol for single-molecule analysis of synaptic protein complex-mediated vesicle recruitment
Source: STAR Protoc. 2025 Dec 5;6(4):104249. doi: 10.1016/j.xpro.2025.104249 (PMC12721264; doi:10.1016/j.xpro.2025.104249)
Supplement: Document S1. Figures S1–S6 [file mmc1.pdf]

Supplementary information

Protocol for single-molecule analysis of synaptic protein complex mediated vesicle recruitment

Akshay Kapadia,[1,2,*] Anne-Sophie Hafner[1,3,]**

[1]Donders Institute for Brain, Cognition and Behavior, Radboud University; Nijmegen, Netherlands.

[2]Technical contact *Correspondence: akshay.kapadia@donders.ru.nl

[3]Lead contact **Correspondence: anne-sophie.hafner@donders.ru.nl

This SI file contains:

Supplementary images depicting primary data (for other tested antibodies/conditions using this experimental protocol) as well as control data and images (artefacts and non-ideal images)

Supplementary images

Figure legends:

Figure S1. Another example of antibody thiolation and validation of capture efficiency, related to preparative step 3, 4 and 14.

(A) Validation of RIM1 antibody reactivity post-thiolation. (i) SDS-PAGE under various reducing and non-reducing conditions showing heavy and light chains detected by Coomassie, Ponceau, and western blot (WB) with anti-rat secondary. N = 2. (ii) Quantification of antibody reactivity by WB and modified ELISA reveals that SATA thiolation (condition 6) did not significantly impair antibody function, and reactivity is preserved after deprotection (condition 7). WB, N = 2, ELISA, N = 3.

(B) RIM1 antibody reactivity in indirect and sandwich ELISA assays. (i–ii) RIM1 antibodies bind to RIM1 protein in synaptosome lysates in a dose-dependent manner, with or without thiolation. Examined using indirect ELISA. (iii) Sandwich ELISA showing capture of Rab3a to RIM1 protein immobilized *via* antibody without (iii; n = 4, N = 2) and with thiolation steps (iv; n = 6, N = 3). Here, the difference in binding and protein capture efficiency indicates RIM1 antibody activity is slightly altered post-modification.

(C) Western immunoblotting images depicting antibody modification (both RIM1 and STX1A) using partial thiolation methods - βME treatment (for different time points) completely abrogated antibody reactivity.

Figure S2. Optimization of surface functionalization with an antibody for SIM-Pull assays, related to preparatory step 6 and 7.

(A) Schematic of surface activation and functionalization strategy. Coverslips are sequentially modified by aminosilanization (APTES), maleimide activation (Sulfo-SMCC), and covalent coupling of thiol-modified antibodies *via* SATA and NH_2OH. The final antibody-conjugated surface is used for vesicle or protein pulldown in the SIM-Pull assay.

(B) TIRF images show the effect of antibody coating concentration and agitation on spot density and uniformity. Surfaces were incubated with increasing concentrations (1, 2 and 5 µg/mL) of antibody without (middle row) or with shaking (bottom row). Activation of the glass surface is necessary for effective functionalization. Insets show higher-magnification views (scale bars: large panels = 10 µm; insets = 0.5 µm). n = 2.

(C) Antibody specificity screen using five different antibodies (AB#1–AB#5) at a fixed concentration (0.5 µg/mL). AB#1–AB#4 show effective surface binding and discrete spot formation. AB#5 yields a low signal, and the no-antibody control shows negligible background. Scale bars: 10 µm. n = 2.

(D) Multichannel TIRF imaging of an ideal AB#1 functionalized surface. Channel 1 (Ch1, 488 channel) shows a robust spot signal from the capture antibody, while Channels 2–4 (Ch2–Ch4, 405, 555, 647 channels, respectively) show minimal or no signal, indicating specificity and minimal crosstalk across channels. Scale bars: left panel = 10 µm; right panels = 0.1 or

0.5 µm. Images are representative of multiple experimental replicates performed during the entirety of this manuscript.

Figure S3. Quantification of particle density and variability across experimental conditions post surface functionalization, related to step 2 and 3.

(A-C) Representative thresholded images (STX1A, STX1, and Syb; data depicted in Fig. 3A-C) used for analyzing particles of immunoisolated proteins labelled with fluorescent antibodies: (A) STX1A only (*red*), (B) STX1A (*red*) and STX1 (*green*), and (C) STX1A (*red*) and Syb (*blue*). Numbers in images indicate percentage of area covered with particles per FOV (mean ± SD). Right, quantification of the number of detected particles per field of view (FOV) from independent experimental replicates. Data are shown as mean ± SD; individual points represent independent measurements from separate FOVs. Scale bars, 5 µm. n = 6, N = 4 (indicated as rep. 1a-b and 2a-b, respectively)

Figure S4. Immunocytochemistry to probe capture-prey (STX1A-Syb) protein complexes tethering SR-SV, related to step 9.

(A) Representative overview images post-ICC showing SR-SV (*magenta*) and STX1A (*red*), and Syb (*green*) channels. Zoomed-in images are depicted in the figure. 5B. N = 3.
(B) Representative overview images post-ICC showing SR-SV (*magenta*) and RIM1 (*red*), and Rab3a (*green*) channels. N = 3.
(C) Zoomed-in panels depicting the tethering of SV show that it is indeed *via* the interaction of RIM1 and Rab3a. The data is representative of three independent experiments.

Figure S5. Representative examples of ideal and non-ideal SV TIRF images, related to step 5 and 6.

Representative images showing synaptic vesicle (SV) preparations labelled with SynaptoRed (SR-SV, *magenta*). Top row: During SV incubation, ideal preparations display well-dispersed SVs (*left*), whereas non-ideal preparations exhibit excessive concentrations and aggregated SVs (*right*). Bottom row: After washing steps, ideal conditions yield well-separated, single tethered SVs (*left*), while non-ideal conditions result in aggregated SVs with larger diameters, which could also result from sterically bound SVs remaining due to reduced washing steps (*right*); scale bars, 5 µm. Please note, all images depicted here are at saturated fluorescence intensities for better visualization.

Figure S6. Representative examples of ideal and non-ideal SV TIRF images post-ICC and washing steps, related to step 5, 6 and 9.

(A) Representative single-molecule TIRF images of SR-SV (*magenta*) showing effects of common preparation errors. Excessive washing can result in SV loss (*left*) or loss of SR fluorescence (*middle*), while the use of a non-isotonic buffer disrupts SV integrity (right), leading to increased background fluorescence. A decrease in fluorescence signals could also be a result of photobleaching the dye due to strong laser power. Scale bars, 5 µm.

(B) Examples of ideal and non-ideal images following ICC and washing. Top row: Ideal images display well-dispersed, intact SVs (*white*) with clear co-localization to capture protein x (*green*). Middle row: Non-ideal but usable images show SVs (SR fluorescence is slightly lost), but images are still suitable for protein localization or quantification (protein y, *blue*). Bottom row: Non-ideal images exhibit improper labelling of protein z (*magenta*), necessitating optimization for ICC labelling of protein. Merged channels are shown in the leftmost panels; SV-only channels in white; protein-specific ICC channels in color. Scale bars, 10 µm (*leftmost panels*) and 0.5 µm (*zoomed panels*).

Figure S1.

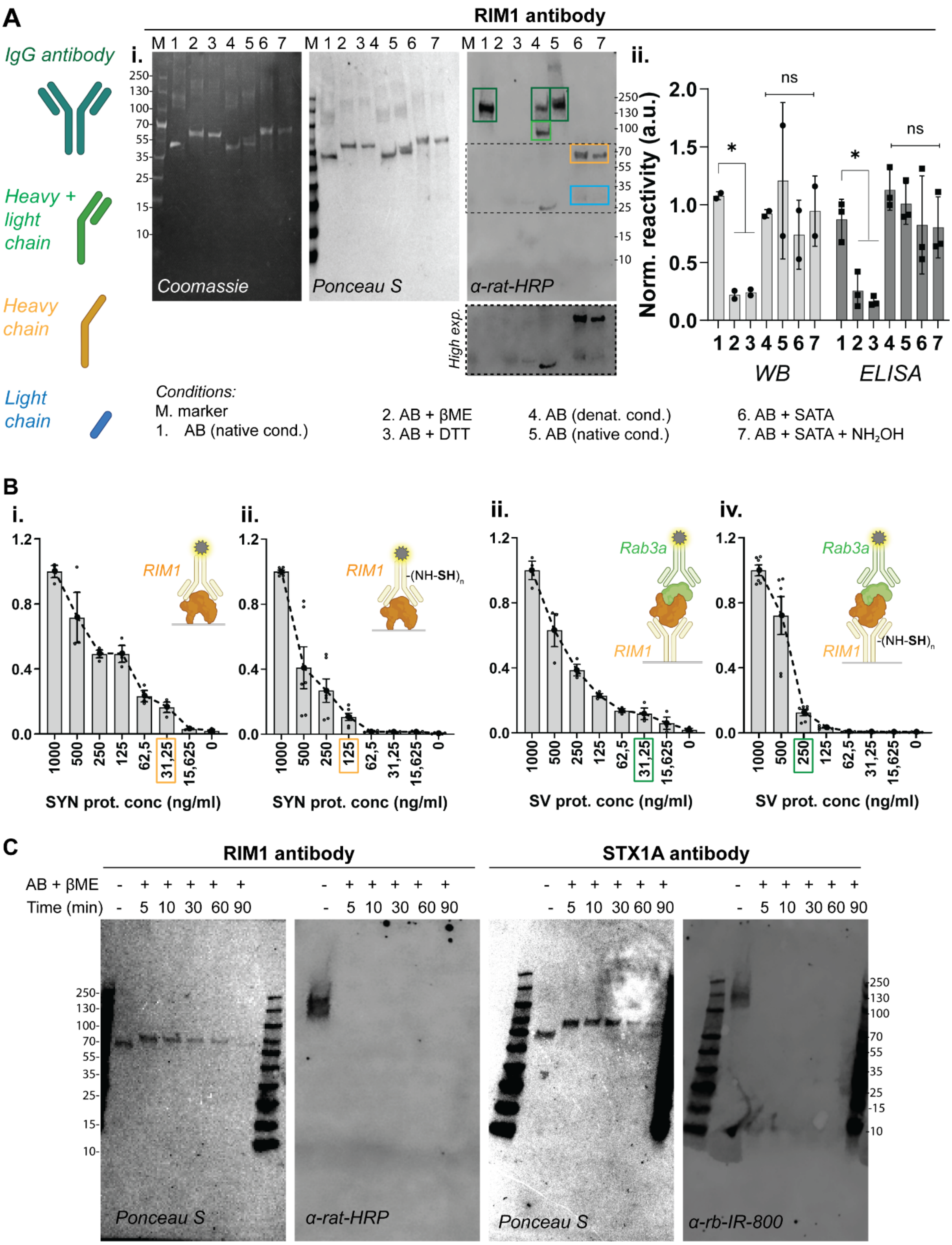

A
RIM1 antibody
IgG antibody
Heavy + light chain
Heavy chain
Light chain
i.
M 1 2 3 4 5 6 7
250- 130- 100- 70- 55- 35- 25- 15- 10-
Coomassie
Ponceau S
α-rat-HRP
High exp.
ii.
Norm. reactivity (a.u.)
2.0 1.5 1.0 0.5 0.0
*
ns
1 2 3 4 5 6 7
WB
ELISA
Conditions:
M. marker
1. AB (native cond.)
2. AB + βME
3. AB + DTT
4. AB (denat. cond.)
5. AB (native cond.)
6. AB + SATA
7. AB + SATA + NH2OH
B
i.
ii.
ii.
iv.
1.2 0.8 0.4 0.0
RIM1
-(NH-SH)n
Rab3a
1000 500 250 125 62,5 31,25 15,625 0
SYN prot. conc (ng/ml)
SV prot. conc (ng/ml)
C
RIM1 antibody
STX1A antibody
AB + βME - + + + + +
Time (min) - 5 10 30 60 90
250- 130- 100- 70- 55- 35- 25- 15- 10-
Ponceau S
α-rat-HRP
α-rb-IR-800

Figure S2.

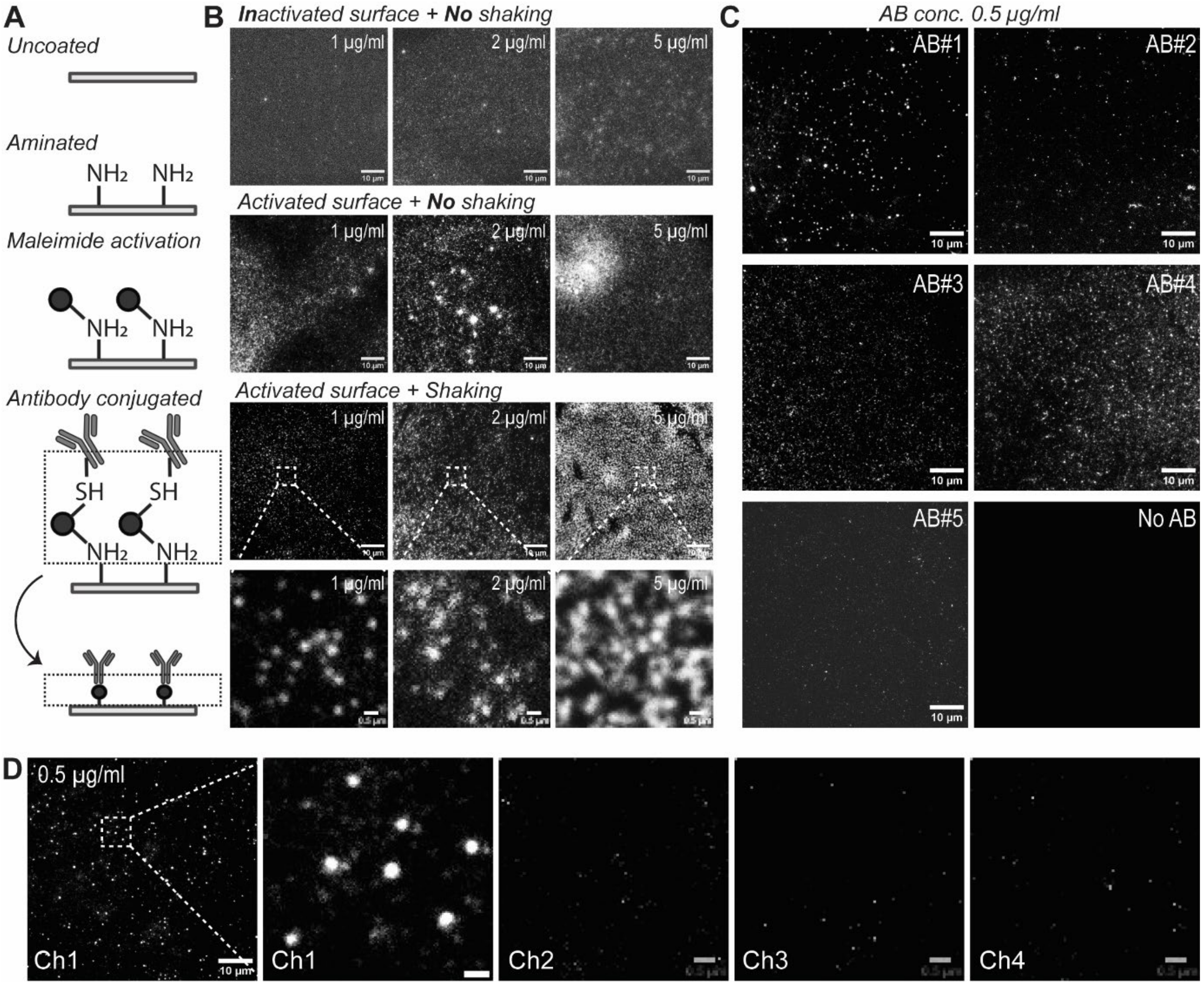

A
Uncoated
Aminated
NH2 NH2
Maleimide activation
Antibody conjugated
SH SH
B
Inactivated surface + No shaking
1 µg/ml
2 µg/ml
5 µg/ml
10 µm
Activated surface + No shaking
Activated surface + Shaking
0.5 µm
C
AB conc. 0.5 µg/ml
AB#1
AB#2
AB#3
AB#4
AB#5
No AB
D
0.5 µg/ml
Ch1
Ch2
Ch3
Ch4

Figure S3.

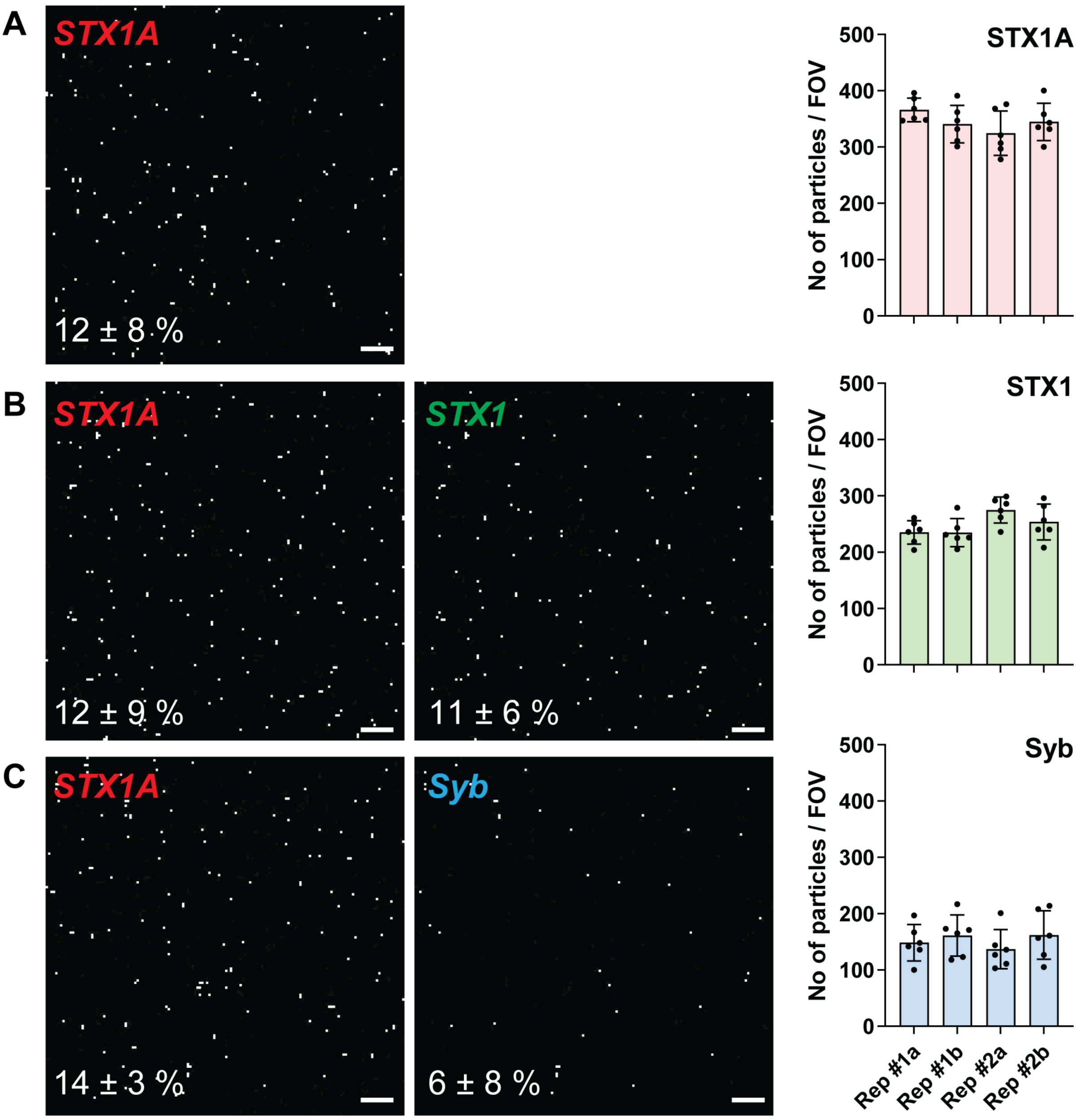

A
STX1A
12 ± 8 %
B
STX1A
12 ± 9 %
STX1
11 ± 6 %
C
STX1A
14 ± 3 %
Syb
6 ± 8 %
STX1A
STX1
Syb
No of particles / FOV
0
100
200
300
400
500
Rep #1a
Rep #1b
Rep #2a
Rep #2b

Figure S4.

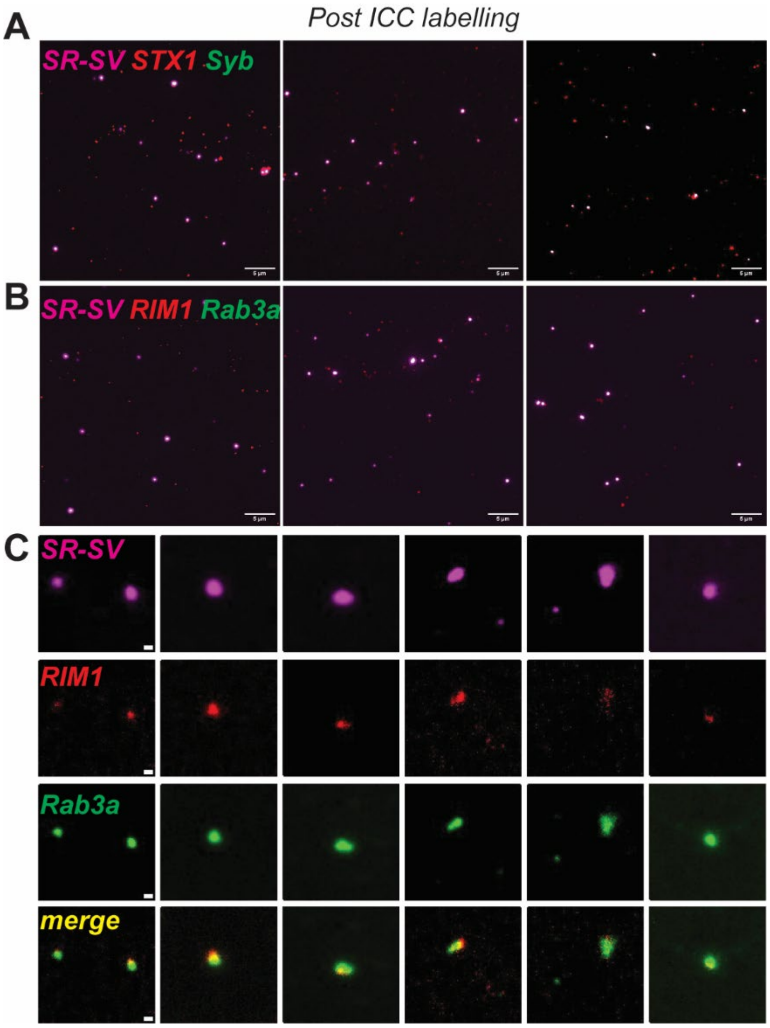

Post ICC labelling
A
SR-SV STX1 Syb
B
SR-SV RIM1 Rab3a
C
SR-SV
RIM1
Rab3a
merge

Figure S5

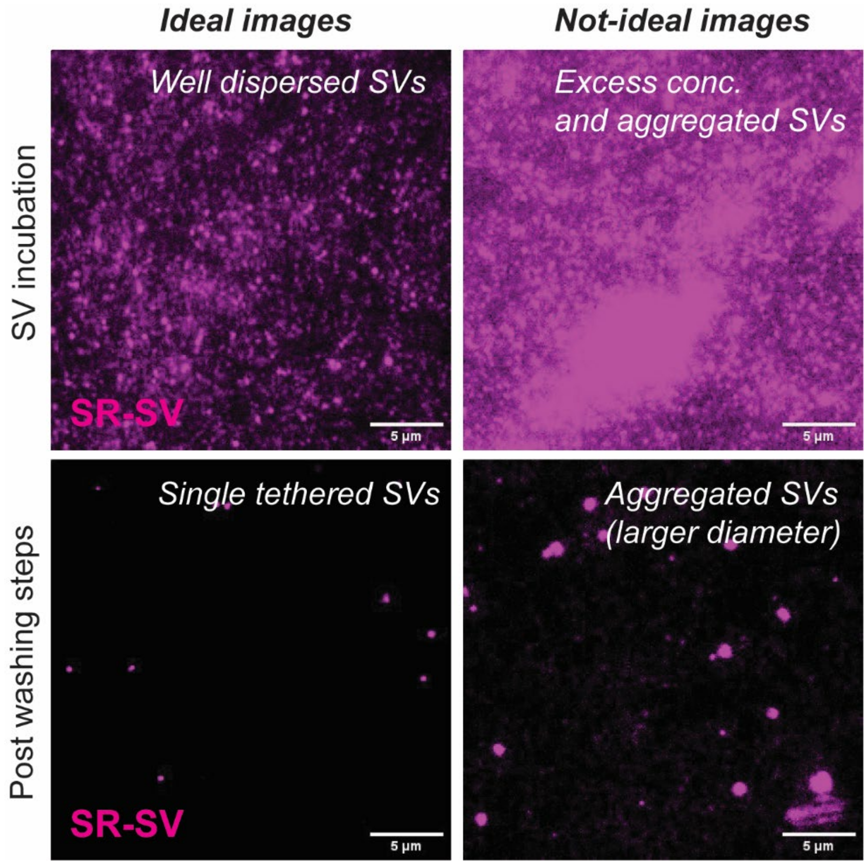
Ideal images
Not-ideal images
SV incubation
Post washing steps
Well dispersed SVs
Excess conc.
and aggregated SVs
Single tethered SVs
Aggregated SVs
(larger diameter)
SR-SV
SR-SV
5 µm
5 µm
5 µm
5 µm

Figure S6.

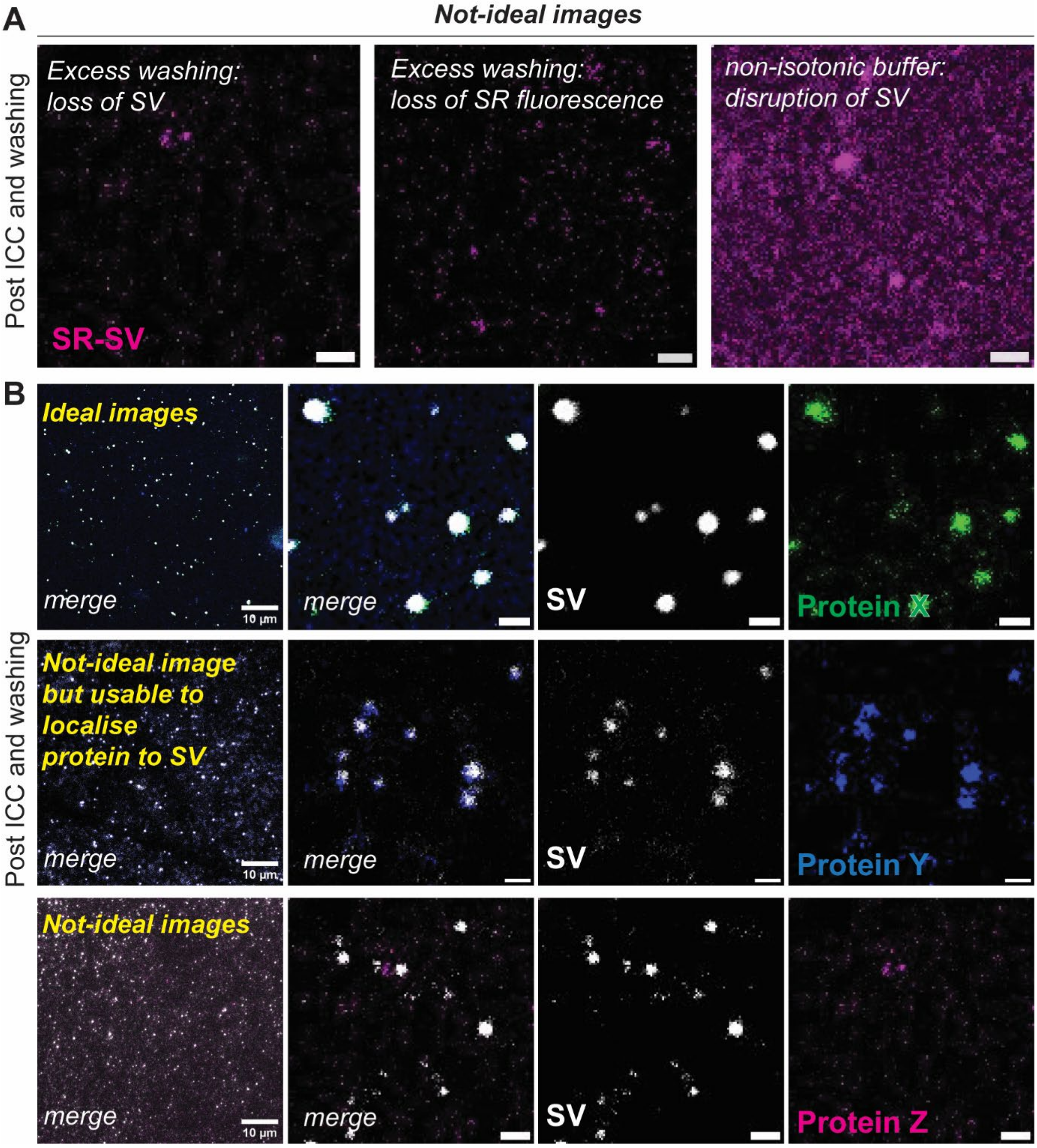

A
Not-ideal images
Post ICC and washing
Excess washing: loss of SV
SR-SV
Excess washing: loss of SR fluorescence
non-isotonic buffer: disruption of SV
B
Ideal images
merge
10 µm
merge
SV
Protein X
Post ICC and washing
Not-ideal image but usable to localise protein to SV
merge
10 µm
merge
SV
Protein Y
Not-ideal images
merge
10 µm
merge
SV
Protein Z
